# Tracking Cefoperazone/Sulbactam Resistance Development *In vivo* in *A. baumannii* Isolated from a Patient with Hospital-Acquired Pneumonia by Whole-Genome Sequencing

**DOI:** 10.3389/fmicb.2016.01268

**Published:** 2016-08-19

**Authors:** Xiaofen Liu, Huajun Zheng, Weipeng Zhang, Zhen Shen, Miao Zhao, Yuancheng Chen, Li Sun, Jun Shi, Jing Zhang

**Affiliations:** ^1^Institute of Antibiotics, Huashan Hospital, Fudan UniversityShanghai, China; ^2^Roche Innovation Center ShanghaiShanghai, China; ^3^Shanghai-MOST Key Laboratory of Health and Disease Genomics, Chinese National Human Genome Center at ShanghaiShanghai, China; ^4^Division of Life Science, The Hong Kong University of Science and TechnologyHong Kong, China; ^5^Key Laboratory of Clinical Pharmacology of Antibiotics, National Population and Family Planning CommissionShanghai, China

**Keywords:** cefoperazone/sulbactam, *Acinetobacter baumannii*, whole-genome sequencing, resistance development, PK/PD indices

## Abstract

Cefoperazone/sulbactam has been shown to be efficacious for the treatment of infections caused by *Acinetobacter baumannii*; however, the mechanism underlying resistance to this synergistic combination is not well understood. In the present study, two *A. baumannii* isolates, AB1845 and AB2092, were isolated from a patient with hospital-acquired pneumonia before and after 20 days of cefoperazone/sulbactam therapy (2:1, 3 g every 8 h with a 1-h infusion). The minimum inhibitory concentration (MIC) of cefoperazone/sulbactam for AB1845 and AB2092 was 16/8 and 128/64 mg/L, respectively. Blood samples were collected on day 4 of the treatment to determine the concentration of cefoperazone and sulbactam. The pharmacokinetic/pharmacodynamic (PK/PD) indices (%T_>MIC_) were calculated to evaluate the dosage regimen and resistance development. The results showed that %T_>MIC_ of cefoperazone and sulbactam was 100% and 34.5% for AB1845, and 0% and 0% for AB2092, respectively. Although there was no available PK/PD target for sulbactam, it was proposed that sulbactam should be administered at higher doses or for prolonged infusion times to achieve better efficacy. To investigate the mechanism of *A. baumannii* resistance to the cefoperazone/sulbactam combination *in vivo*, whole-genome sequencing of these two isolates was further performed. The sequencing results showed that 97.6% of the genome sequences were identical and 33 non-synonymous mutations were detected between AB1845 and AB2092. The only difference of these two isolates was showed in sequencing coverage comparison. There was a 6-kb amplified DNA fragment which was three times higher in AB2092, compared with AB1845. The amplified DNA fragment containing the *bla*_OXA-23_ gene on transposon *Tn2009*. Further quantitative real-time PCR results demonstrated that gene expression at the mRNA level of *bla*_OXA-23_ was >5 times higher in AB2092 than in AB1845. These results suggested that the *bla*_OXA-23_ gene had higher expression level in AB2092 via gene amplification and following transcription. Because gene amplification plays a critical role in antibiotic resistance in many bacteria, it is very likely that the *bla*_OXA-23_ amplification results in the development of cefoperazone/sulbactam resistance *in vivo*.

## Introduction

*Acinetobacter baumannii* is a non-fermentative, gram-negative opportunistic pathogen that can cause hospital-acquired pneumonia (HAP), blood infections and urinary tract infection, among others (Perez et al., 2007). The rapid spread ofthe multi-drug-resistant *A. baumannii* has resulted in very limited therapeutic options in the clinic. Sulbactam, a β-lactamase inhibitor, has intrinsic antimicrobial effects against *A. baumannii* by binding to penicillin-binding protein 2 (Peleg et al., 2008). It is commercially available in a combined formulation of ampicillin and cefoperazone. The clinical efficacy of cefoperazone/sulbactam has been shown in previous work (Xin et al., 2013; Sipahi et al., 2014; Xia et al., 2014). This combination has recently been applied to treat critically ill patients receiving continuous venovenous hemofiltration, and 11 of 14 patients survived (Gao et al., 2016). This combination was also administrated to treat neurosurgical patients in a pilot study, with the results showing that cerebrospinal fluid penetration of cefoperazone/sulbactam could be enhanced after neurosurgical impairment of the blood-brain barrier (Wang et al., 2015). In a retrospective review of the outcomes for patients with cefoperazone/sulbactam treated *A. baumannii* bacteremia, 77% of the patients (27/35) presented successful clinical efficacy (Choi et al., 2006). Because the combination has been widely used in the clinic, the resistance rate was monitored and observed to increase from 25.0 to 37.7% from 2004 to 2010 in China (Hu et al., 2016). However, the underlying mechanism is not well understood *in vitro* or *in vivo*, for sulbactam alone or the combination. Sulbactam alone has been reported to cause resistance in *A. baumannii* via PBP3 mutation *in vitro*, despite a lack of information regarding natural pbp3 mutations in clinical isolates (Penwell et al., 2015). The detection and expression of *bla*_TEM-1_ has been suggested to relate to the minimum inhibitory concentration (MIC) of sulbactam in *A. baumannii* (Waltner-Toews et al., 2011). The combination resistance in *Klebsiella pneumoniae* in an *in vitro* study was reported owing to two different mechanisms, loss of a 39-kDa outer membrane protein and presence of TEM-2 β-lactamase (Rice et al., 1993).

Whole-genome sequencing is powerful and can reveal a vast amount of DNA information from a global perspective. The published complete genome sequences for many bacteria are beneficial and efficient for strain-to-reference sequencing and bioinformatics analysis (Mardis, 2008). They can be used to identify resistance genes as well as minor changes in the genome due to mutation, gene transfer, gene duplication, and amplification. It has been seen as a basis for whole-genome sequencing revealing the mechanism of bacterial pathogen resistance that develops in patients (Snitkin et al., 2011; Wright et al., 2014; Holt et al., 2015). Wright et al. (2016) sequenced 136 strains of *A. baumannii* isolated from patients and showed the genome changes occurred mainly due to the single nucleotide variance in protein coding regions and IS element. Genome sequenced *A. baumannii* showed the colistin resistance was due to the mutations in transcriptional regulatory genes (Cheah et al., 2016). Susceptible *S. aureus* evolved resistance via a 35-point mutation in 31 loci in a patient with a bloodstream infection receiving vancomycin therapy (Mwangi et al., 2007). Tigecycline resistance to *A. baumannii* has been reported to be due to the deletion of three contigs *in vivo* (Hornsey et al., 2011). Additionally, gene amplification was frequently detected due to the antibiotic pressure. The evolution of β-lactamase resistance was studied by exposing *Salmonella typhimurium*, which contain low level β-lactamase resistance, in an environment of progressively increasing concentrations of cephalosporin. The results showed an amplification of *bla*_TEM-1_ gene copy number followed by acquisition of gene mutations (Sun et al., 2009). A genome-wide assay of *E. coli* strain in 78 different antibiotic environments found that 56 genes were amplified to fit the environment and reproducible increasing in MIC for their corresponding antibiotics was detected (Soo et al., 2011). The amplification of aminoglycoside resistance gene *aphA1* in *A. baumannii* has been considered to be the mechanism underlying the development of tobramycin *in vivo* (McGann et al., 2014).

In the present study, two *A. baumannii* isolates, AB1845 and AB2092, were collected before and after cefoperazone/sulbactam therapy from a patient with HAP. Pharmacokinetics/pharmacodynamics (PK/PD) was applied to evaluate the dosage regimen and development of resistance. Moreover, whole-genome sequencing was employed to investigate the mechanism of cefoperazone/sulbactam resistance of the two isolates developed *in vivo*.

## Materials and Methods

### Medical Records and Treatment

This study was approved by the institutional review board of Huashan Hospital affiliated to Fudan University. Written informed consent was obtained from the patient. A 19-year-old male patient with serious brain trauma was admitted to Huashan Hospital (Shanghai, China). The patient had a high fever with white blood cell counts out of the normal range after being checked into bed for 48 h. Both chest X-Ray radiophotography and computed tomography showed pneumonia. *A. baumannii* (AB1845) was isolated from the sputum of the patient, revealing bacterial infection. Hence, the patient was treated with meropenem 0.5 g q8h without clinical efficacy. On day 6, the dosage regimen was changed to cefoperazone/sulbactam (2:1, 3 g every 8 h with a 1-h infusion, SULPERAZON^®^, batch no. 95839116) for 20 days. The treatment was stopped when body temperature and white blood cell counts returned to the normal range; additionally, *A. baumannii* was not detected in the sputum. However, the patient had a fever, and *A. baumannii* (AB2092) was again isolated 4 days after cefoperazone/sulbactam treatment. The cefoperazone/sulbactam treatment was finally considered to have failed both clinically and microbiologically efficacy.

### Pharmacokinetics of Cefoperazone/Sulbactam Treatment

Blood samples were collected before the first infusion, immediately after the infusion (0 h), and at 0.5, 1, 2, 4, 6 h afterward on day 4 (i.e., steady state) of the cefoperazone/sulbactam treatment. The drug concentration was determined using the LC-MS/MS method (Zhou et al., 2010), which was validated according to the CFDA guidelines on bioanalytical method validation. The pharmacokinetic parameters of the patients were calculated by Winnonlin (v6.0, phoenix). The patient’s pharmacokinetic profiles of cefoperazone and sulbactam were fit into a well-developed population PK model (Chen et al., 2015). The PK/PD indice of cefoperazone and sulbactam was %T_>MIC_, namely the percentage of the dosing interval for which the plasma concentration of cefoperazone or sulbactam above the MIC. It was calculated based on the drug pharmacokinetic profile and MICs of the combination (Lips et al., 2014).

### Bacterial Isolates and Susceptibility Testing

*Acinetobacter baumannii* AB1845 and AB2092 were isolated from the sputum of the patient before and after cefoperazone/sulbactam therapy. Both isolates were stored at -80°C. The MIC was determined in cation-adjusted Mueller-Hinton broth (Becton-Dickinson, Sparks, MD, USA) using a broth microdilution according to Clinical and Laboratory Standards Institute (CLSI) standards. The MICs of the β-lactam/β-lactamase inhibitor combination, including cefoperazone/sulbactam, carbapenems, colistin, and polymyxin B, were determined.

### Genome Sequencing and Analysis

Genomic DNA was extracted using a Genomic DNA Purification Kit (Tiangen, Beijing, China) according to the instruction manual and stored at -80°C before sequencing. A 300-bp paired-end library was constructed for the purified DNA sample following the standard Illumina paired-end protocol. Cluster generation was performed in C-bot, and sequencing was performed on the Illumina Hiseq2500 with 150 cycles. The sequence reads were cleaned using the FASTX toolkit^1^. Genome assembly was performed using Velvet (Ver 1.0.15) (Zerbino and Birney, 2008). Putative protein-coding sequences were determined by combining the prediction results of the glimmer 3.02 (Delcher et al., 2007) and Z-Curve (Guo and Zhang, 2006) program. The phylogenetic tree was constructed using PHYML (Guindon and Gascuel, 2003) by concatenating orthologs based on the protein sequences in the published genome. Functional annotation of CDS was performed by searching the NCBI non-redundant protein database and the KEGG protein database (Kanehisa et al., 2016). Gene annotation was mapped to the *A. baumannii* MDR-ZJ06 genome (from the phylogenetic tree) using Bowtie2 with default settings (Langmead and Salzberg, 2012). Single Nucleotide Polymorphisms (SNPs) from alignments were called using Samtools-0.1.16 (Li et al., 2009), and the output was generated in the pileup format. Gene amplification was detected by comparing the coverage of the reads in AB1845 and Ab2092 using the read depth method (Redon et al., 2006).

### Quantitative Real-Time PCR Analysis of *bla*_OXA-23_

The mRNA expression level of *bla*_OXA-23_ was quantified by real-time PCR. AB1845 and AB2092 were cultured in cation-adjusted Mueller-Hinton broth and collected during the early log phase. Total RNA was extracted using a TaKaRa Mini BEST Universal RNA Extraction Kit (TaKaRa Biotechnology, Dalian, China) according to the manufacturer’s instruction. Quantitative real-time PCR (qPCR) was performed using a two-step process. RNA was first reverse-transcribed to cDNA (TaKaRa Biotechnology, Dalian, China), and real-time PCR was conducted on an ABI Vii7 (Applied Biosystems, Carlsbad, CA, USA) using a TaKaRa SYBR^®^ FAST qPCR Kit with 40 cycles of denaturation for 5 s at 95°C, annealing for 30 s at 50°C, and extension for 20 s at 72°C. The PCR primers for the *bla*_OXA-23_ gene were F: CCGAGTCAGATTGTTCAAGGA and R: TGTAGAGGCTGGCACATATTC; and those for 16S rRNA were F: GGCGGCTTATTAAGTCGGATG and R: TTCGTACCTCAGCGTCAGTATT. The melting curve analysis was performed immediately after amplification to verify the specificity of the PCR amplification products.

Fluorescence was measured at the end of the annealing-extension phase of each cycle. A threshold value for the fluorescence of all samples was set manually. The reaction cycle at which the PCR product exceeded this fluorescence threshold was identified as the threshold cycle (CT). The relative quantitation was calculated by the 2-^ΔΔCT^ method (Schmittgen and Livak, 2008). The student *t*-test was applied to evaluate the significance of the gene expression level of the two isolates.

## Results

### Antibiotic Susceptibility

Minimum inhibitory concentrations were obtained for cefoperazone/sulbactam combinations, carbapenems, colistin and polymyxin B for the pre-therapy isolate AB1845 and the post-therapy isolate AB2092 (**Table 1**). The MICs of the cefoperazone/sulbactam combination were 16/8 mg/L for AB1845; and the MICs of mono cefoperazone and sulbactam were >128 mg/L and 32 mg/L. The results supported a synergistic effect of the combination (FICI < 0.5, MIC of cefoperazone was set at 128 mg/L for the FICI calculation). However, the MICs of the combination and mono drugs were 128/64, >128, and 128 mg/L for AB2092. The combination did not display a synergistic effect on AB2092.

**Table 1:** The minimum inhibitory concentrations (MICs) of different antibiotics for AB1845 and AB2092.

Antibiotics	MIC (mg/L)	
	
	AB1845	AB2092
**Cefoperazone/sulbactam**	**16/8^∗^**	**128/64**
Cefoperazone	>128	>128
Sulbactam	32	128
**Meropenem**	**16**	**64**
**Imipenem**	**16**	**64**
**Doripenem**	**32**	**128**
Colistin	2	2
Polymyxin B	1	1


*^∗^FICI = 0.375; FICI = MIC_*(A)*_/MIC_*(A in combination)*_ + MIC_*(B)*_/MIC_*(B in combination)*_. The bold values showed the increasing MIC of AB1845 compared with AB2092.*

Interestingly, AB1845 and AB2092 also developed meropenem resistance with MIC increased from 16 to 64 mg/L, although the bacteria only exposed to meropenem for a short term (0.5 g every 8 h for 4 days). The isolates also developed resistance to imipenem and doripenem. Since both cefoperazone and meropenem are β-lactam antibiotics, the increased MIC in both cefoperazone/sulbactam and carbapenems may imply the similar underlying resistance mechanism. The isolates were only sensitive to colistin and polymyxin B, indicating an alternative choice for the AB2092 treatment. The MICs of the antibiotics are shown in **Table 1**.

### Pharmacokinetics and Pharmacodynamics (PK/PD)

**FIGURE 1:**
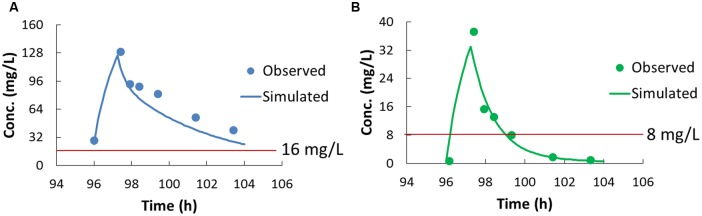
**The pharmacokinetic profiles of cefoperazone **(A)** and sulbactam **(B)**.** The dots in **(A,B)** are the concentration determined by LC-MS/MS (before the first infusion on Day 4 and 0.5, 1, 2, 4, 6 h after the infusion), and the curves are the profiles fitted to the population pharmacokinetic model. The red lines were indicated the concentration for the MIC of AB1845.

Plasma concentrations of cefoperazone and sulbactam at steady state were determined using the validated LC-MS/MS method, and the data were fitted into a developed population PK model (**Figure 1**). The pharmacokinetic parameters of cefoperazone and sulbactam are summarized in **Table 2**. The T_1/2_ of cefoperazone was 4.1 h, which was a bit longer than the reported 1.8 h (Reitberg et al., 1988). This could attribute to liver dysfunction (Boscia et al., 1983) according to the medical records of the patients. The T_1/2_ of sulbactam was 1.3 h, which was comparable to the published data (Reitberg et al., 1988).

Pharmacokinetic/pharmacodynamic indices were calculated to evaluate the drug dosage regimen. The %T_>MIC_ was the most predictive PK/PD index for cefoperazone and sulbactam (Cooper et al., 2011; Sy et al., 2015), and the MIC of the combination was used for the %T_>MIC_ calculation. In this study, the %T_>MIC_ was 100% for cefoperazone and 34.5% for sulbactam for AB1845; and the %T_>MIC_ was 0% for both cefoperazone and sulbactam for AB2092.

**Table 2:** Pharmacokinetic parameters of cefoperazone and sulbactam.

Parameters	Units	Cefoperazone	Sulbactam
*T*_1/2_	h	4.1	1.3
*T*_max_	h	1.4	1.3
*C*_max_	mg/L	129.6	37.2
AUC_0-8_	mg h/L	529.1	66.5
AUC_0-inf_	mg h/L	766.0	68.3
Vd	L	15.5	26.6
CLt	L/h	2.6	14.6
MRT_0-8_	h	2.6	1.5
MRT_0-inf_	h	5.7	1.6


### Whole-Genome Sequencing of AB1845 and AB2092

The two isolates were initially typed by PFGE of *ApaI*-digested genomic DNA and shown to be the same strain. Whole-genome sequencing produced 12,050,346 and 12,191,576 pairs of 150-bp reads for AB1845 and AB2092, respectively. Assembly of the AB1845 and AB2092 genomes resulted in 132 and 131 contigs larger than 500 bp, comprising 4.0 megabases of sequence and representing a median 930-fold coverage. The AB1845 draft assembly has been deposited in GenBank (accession number LVYA00000000), and raw sequence reads for AB1845 and AB2092 have been submitted to NCBI’s Sequence Read Archive under the study accession number SRP072783.

**FIGURE 2:**
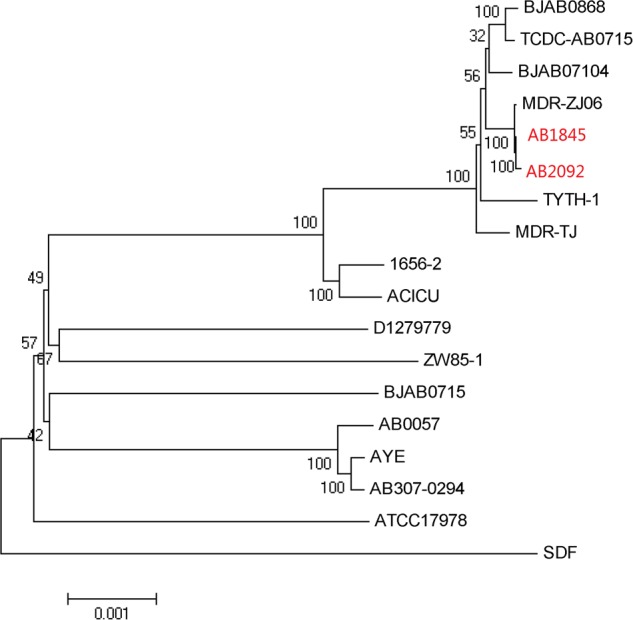
**Phylogenetic tree of AB1845 and AB2092 with 16 complete genomes of *A. baumannii* isolates in ftp://ftp.ncbi.nlm.nih.gov/genomes/archive/old_genbank/**.

**FIGURE 3:**
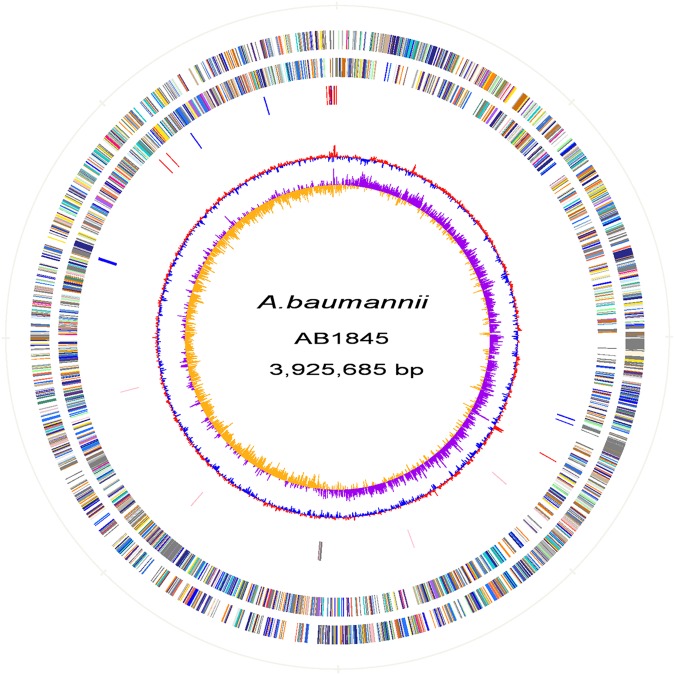
**Atlas of the *A. baumannii* AB1845 draft genome.** Each concentric circle represents the genomic data for AB1845. The two outer circles illustrate the predicted coding sequences on the plus and minus strands, respectively, colored by functional categories according to the COG classification. The third circle represents the location of the nucleotide substitution between AB1845 and AB2092, with blue representing genes affected by synonymous Single Nucleotide Polymorphisms (SNPs) and red representing SNPs in the intergenic region. The fourth circle displays the loci of the β-lactamase genes (pink) and genes around *bla*_OXA-23_ (gray). The fifth circle shows the GC content, and the 6th circle (innermost) represents GC skew (G–C)/(G+C) calculated using a 1-kb window.

Automated gene prediction detected 3,811 and 3,810 putative coding sequences (CDSs) for AB1845 and AB2092, respectively. A phylogenetic tree was constructed, and the genome similarity was compared (**Figure 2**). The 3,718 CDSs were homologous (defined as a BLASTN *e*-value ≤ 1e^-10^) to the genome of *A. baumannii* MDR-ZJ06, which was a multi-drug-resistant isolate belonging to International clone II and wide spreading in China (Zhou et al., 2011). MDR-ZJ06 was used as a reference genome for annotating genes in AB1845 and AB2092 (**Figure 3**). The results showed that the two isolates possess 97.6% similarity to MDR-ZJ06, with 921 and 915 SNPs, respectively. Certain genes were missing in both AB1845 and AB2092 compared with MDR-ZJ06. For example, 17 genes had a 15-kb fragment that was missing in the resistant island *Aba*R22, which includes transposition helper proteins, site-specific tyrosine recombinase, transposase protein A and conserved hypothetical proteins, among others.

The CDS of AB1845 was also mapped to the published plasmid sequence. It showed that certain AB1845 genes mapped to plasmid ABKp1 from the *A. baumannii* 1656-2 plasmid with 97.6% similarity.

A comparison of the CDS of AB1845 and AB2092 revealed only 33 SNPs. All of these SNPs were synonymous, indicating that SNP was not the main reason for the development of resistance (**Supplementary Table S1**).

### β-Lactamase and Gene Amplification

A well-known resistance enzyme for β-lactam antibiotics is β-lactamase. The class A, C, and D classes (*bla*_OXA-23_ and *bla*_OXA-51_) β-lactamase were identified in both AB1845 and AB2092 (**Supplementary Table S2**).

**FIGURE 4:**
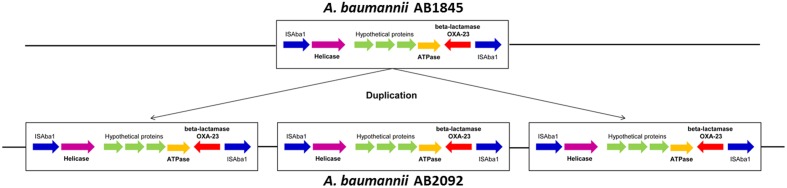
**The illustration of the 6-kb DNA fragment of *Tn2009* in AB1845 and AB2092.** The fragment was amplified three times in AB2092.

Gene amplification was detected by whole-genome sequencing in combination with the read depth method (Cantsilieris et al., 2012). Approximately 99.9% of the genes had a coverage ratio of AB2092 to AB1845 of approximately 1.0, but six genes (out of 3871 genes) had a coverage ratio over 3.0 (**Supplementary Table S3**). These six genes were AAA ATPase, DEAD/DEAH box helicases, *bla*_OXA-23_, and three hypothetical genes. Among these genes, AAA ATPase and DEAD/DEAH box helicases are involved in the function of DNA replication, repair, transcription, and two uncharacterized genes (Johnson and Mckay, 1999; Iyer et al., 2004). The *bla*_OXA-23_ was gene encoded class D β-lactamase and conferred resistance to carbapenems and cefoperazone (Pascale and Wright, 2010; Bush, 2013). All six genes were located in a 6-kb sequence fragment in AB1845 designated as *Tn2009*. *Tn2009* was flanked by two *ISAba1* elements that contributed to the duplication of *Tn2009* in AB2902 (Liu et al., 2015). The amplified genes were transcribed in the same orientation (**Figure 4**).

### mRNA Level of *bla*_OXA-23_ in AB1845 and AB2092

**FIGURE 5:**
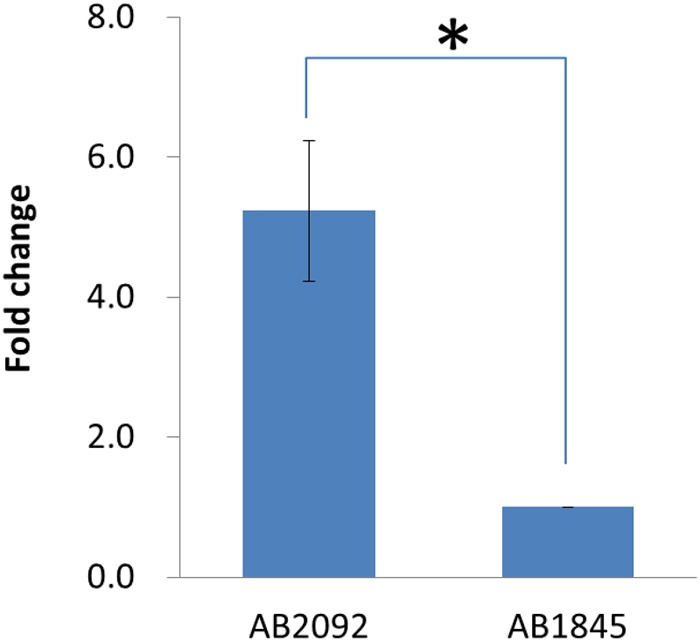
**Quantitative real-time PCR results for *bla*_OXA-23_ in AB1845 and AB2092 (^∗^student *t*-test *p* < 0.05)**.

Quantitative real-time PCR was conducted to measure the mRNA levels of *bla*_OXA-23_ in AB1845 and AB2092. Both isolates were collected at the early log phase, and the qPCR results showed that the mRNA level of *bla*_OXA-23_ was five times higher in AB2092 than in AB1845 (**Figure 5**). This finding was consistent with the gene amplification results.

## Discussion

*Acinetobacter baumannii* commonly colonizes in respiratory tract of the hospitalized patients (Peleg et al., 2008). It is difficult to characterize *A. baumannii* isolated from sputum belongs to the upper airway colonization or the causative pathogen of pneumonia. In this study, the two isolates were considered causative pathogens, as verified by pneumonia testing and symptoms such as cough, fever, and high white blood cell counts. To examine the mechanism of resistance development during cefoperazone/sulbactam treatment, both PK/PD indices and genomic sequencing were employed to understand the problem from a PK/PD and molecular perspective.

Exposure to a sub-optimal antibiotic concentration is the most important factor in the development of bacterial resistance. To optimize the antibiotic dosage regimen and prevent resistance, the PK/PD which bridges drug exposure and effect, has been widely applied in the clinics (Abdulaziz et al., 2015; Asín-Prieto et al., 2015; Monogue et al., 2015). To date, the PK/PD helps adjust dosage based on the evaluation of steady-state drug concentrations (Martinez et al., 2012). In this study, blood samples were collected at steady state, and the MIC of the bacteria was determined. As the most predictive PK/PD index, the %T_>MIC_ of cefoperazone and sulbactam was calculated and compared with published PK/PD targets. The published target %T_>MIC_ of β-lactam antibiotics was 40–70% (Drusano, 2004); however, there was no published target of sulbactam because of a lack of pharmacodynamic data. In our present dosage regimen (3 g q8h, infusion for 1 h), the target was 100% of cefoperazone and 34.5% of sulbactam for AB1845. Cefoperazone clearly reached its target, whereas there were no criteria for sulbactam. In a study of PK/PD simulation for sulbactam in healthy volunteers, in order for *A. baumannii* with an MIC of 8 mg/L to achieve the target of 40%, the sulbactam had to be administered as a 4-h infusion of 3 g q8h (Jaruratanasirikul et al., 2013). It has even been suggested that the daily dose of sulbactam can be administered up to 6 g for *A. baumannii* infection (Argyres, 2010) to achieve enhanced efficacy. A comparison of these data revealed that sulbactam should be administered at a much higher dose or prolonged infusion time. A suboptimal dose of sulbactam could lead to the development of resistance during treatment. Additional research to identify the PK/PD target of sulbactam specific to *Acinetobacter* is needed.

Whole-genome sequencing was further employed to investigate the mechanism of resistance from a molecular perspective. Comparison of the genome between AB1845 and AB2092 revealed no gene deletions or mutations (only 33 synonymous SNPs). However, the reads depth method showed six amplified genes on *Tn2009*, including *bla*_OXA-23_. The *bla*_OXA-23_ gene encodes the class D β-lactamase which confers resistance to the last resort carbapenem antibiotics. The OXA-23-producing *A. baumannii* is widely disseminated in multi-drug-resistant *A. baumannii*. Although the *bla*_OXA-23_ gene has been mainly identified on plasmids, its chromosomal location has also been reported (Zhou et al., 2011). The transportable elements, *Tn2006*, *Tn2007*, *Tn2008*, and *Tn2009*, play a key role in the transfer of the *bla*_OXA-23_ gene to different locations in one bacterium or to different isolates (Corvec et al., 2007; Liu et al., 2015). *Tn2008* and *Tn2009* have mostly been associated with the transfer of *bla*_OXA-23_ in China (Adams-Haduch et al., 2008; Zhou et al., 2011; Liu et al., 2015). IS elements are associated with the genome gain/loss of genes, especially resistant genes. These two transposons share IS*Aba1* upstream from *bla*_OXA-23_, which belongs to the IS4 family, provides promoter for *bla*_OXA-23_ (Turton et al., 2006; Mugnier et al., 2009), which could explain the gene duplication or amplification in this study. The mRNA level of *bla*_OXA-23_ confirmed the higher expression level of AB2092 and AB1845. There is evidence that gene duplication and amplification in bacteria was directly associated with adaptation to environmental changes, including antibiotic stress (Sandegren and Andersson, 2009). In a study testing the relationships of gene amplification and β-lactams resistance increase showed that 70% colonies of *S. Typhimurium* with increased resistance to cephalosporin had increased *bla*_TEM_ gene copy numbers (Sun et al., 2009). Recent study showed the heteroresistance of *E. Coli* and *S. Typhimurium* was due to the amplification of *pmrD* gene which encoded a protein modifying lipid A. Moreover, the heteroresistance phenotype is associated with different copy numbers of the *pmrD* gene (Hjort et al., 2016). Numerous studies have reported gene duplication and amplification related to bacterial resistance to antibiotics *in vitro* (Bertini et al., 2007). During the macrolide treatment of a *Streptococcus pneumoniae* infection, the bacterium developed resistance due to an 18 bp duplication in the *rplV* gene, leading to the treatment failure. The duplication of *rplV* gene led to the blocking of macrolide binding site on ribosome (Musher et al., 2002). Another example of gene amplification in bacterial isolates from human patients involves the *Streptococcus agalactiae*, a leading cause of neonatal infection. The amplification of a four-fold tandemly amplified 13.5-kbp region and a 92 kbp duplication conferred drug resistance to both sulfonamide and trimethoprim (Brochet et al., 2008).

In this study, the isolates developed resistance to cefoperazone/sulbactam as well as carbapenem antibiotics. The whole-genome sequencing showed the amplified *bla*_OXA-23_ in the isolates is very likely the contribution to the resistance. It is rational to conferred resistance to carbapenem antibiotics by the amplification of *bla*_OXA-23_. However, this is the first time to report the amplification of *bla*_OXA-23_ results in cefoperazone/sulbactam combination resistance. Further studies will be focused on collecting more clinical isolates pairs of *A. baumannii* to support the mechanism proposed.

## Author Contributions

XL designed the genomic sequencing experiments. XL and HZ performed the data analysis. XL and ZS conducted the qPCR experiments. YC conducted the pharmacokinetic parameter calculation. LS extracted the genomic DNA for sequencing. MZ conducted the MIC test and the PFGE test. JS and JZ conceived of the study and devised the overall study strategy. XL wrote the manuscript with input from all authors.

## Conflict of Interest Statement

The authors declare that the research was conducted in the absence of any commercial or financial relationships that could be construed as a potential conflict of interest.

## Footnotes

1 http://hannonlab.cshl.edu/fastx_toolkit/
